# How I do it: modified lichtenberger-brown tracheoesophageal puncture procedure

**DOI:** 10.1186/s40463-022-00571-z

**Published:** 2022-06-06

**Authors:** Nikolay R. Sapundzhiev, Asen G. Asenov, Blagovesta Spasova, Petya S. Genova, Georgi I. Davidov, Darina Ivanova

**Affiliations:** 1 Division of Otorhinolaryngology, Department of Neurosurgery and ENT Diseases, Medical University “Prof. Dr. P. Stoyanov”, 55, Marin Drinov Str., 9002 Varna, Bulgaria; 2 Department of Otolaryngology, MBAL Plovdiv Hospital, Plovdiv, Bulgaria; 3 Department of Diagnostic Imaging and Radiotherapy, Medical University “Prof. Dr. P. Stoyanov”, Varna, Bulgaria

**Keywords:** Laryngectomy, Alaryngeal speech, Tracheoesophageal puncture

## Abstract

**Background:**

Tracheoesophageal puncture (TEP) with use of a prosthesis is nowadays a standard for voice restoration after laryngectomy. Different TEP approaches exist.

**Methods:**

We retrospectively reviewed our series of patients who underwent TEP by a novel technique, based partially on the Lichtenberger endo-extralaryngeal needle carrier. The instrument is covered with a protective Nelaton catheter and introduced via the mouth to the neopharynx/esophagus. No rigid endoscope is used for visualization of the TEP site. The tip is palpated through the stoma at the posterior tracheal wall and incision is done to the catheter tip. The prosthesis is introduced through the mouth and the neopharynx in a retrograde fashion.

**Results:**

In 14 laryngectomees with postoperative radiation voice prosthesis was successfully placed with this technique. A total of 18 procedures were performed. One misplacement occurred. No other early or late complications were observed or any other TEP or prosthesis related problems.

**Conclusions:**

The rationale of our technique is to simplify the procedure, avoid risk-bearing approaches and instruments such as rigid endoscopes, simplify the armamentarium and reduce tissue trauma. The initial clinical experience in 18 TEPs confirmed it usefulness in both standard and anatomically challenging situations.

***Trial registration*:**

The current study obtained the ethical approval from the Faculty of Medicine at Medical University "Prof. Dr. Paraskev Stoyanov"—Varna, Bulgaria (Protocol 087/24.10.2019 (retrospectively registered).

**Graphical abstract:**

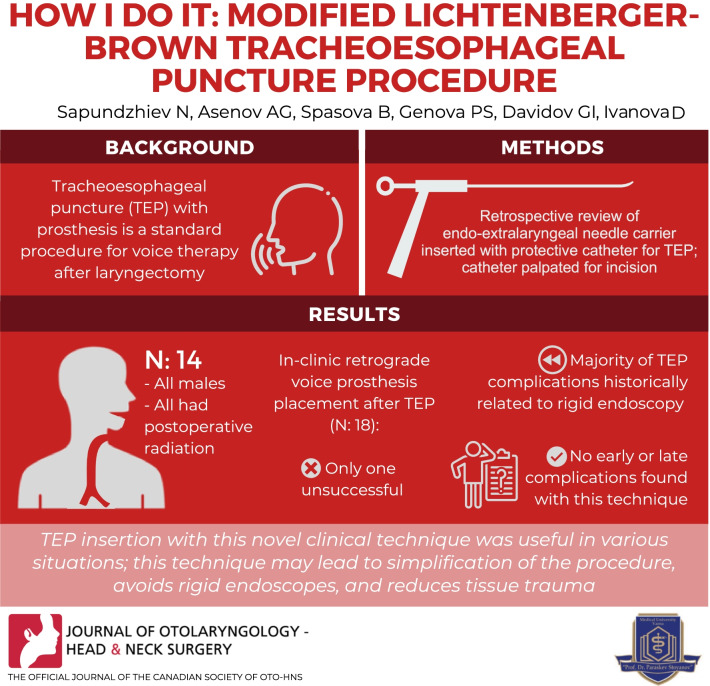

## Background

Nowadays tracheoesophageal puncture (TEP) with use of a prosthesis as introduced by Singer and Blom is the primary option of choice for voice restoration in laryngectomees [1]. Of all variations of this puncturing technique, summarized in Table 1, special attention deserves the endo-extra esophagotracheal approach for secondary TEP, advocated by Lichtenberger [2]. An intricate system, based on the original endo-extralaryngeal needle carrier (EENC), allows for the puncture be performed from the anterior wall of the esophagus into the posterior wall of the trachea, thus eliminating the risk for injuring the back wall of the pharynx. However, this technique is not without its problems, despite the belief of its ease and safety in execution. In patients with stiff tissues at the site of TEP we have previously experienced problems already when pushing the needle and especially when the cone was perforating. Sometimes excessive pull on the thread was necessary to bring out the cone with the catheter. Evaluating our previous experience and the mentioned difficulties we have met with this technique, we developed a hybrid approach, which proves to be easier, appears safer and more versatile.

**Table 1:** Comparison of technical characteristics and initial result of different approaches for secondary TEP

Author	n	Visualization at TEP site via	Determination of TEP position	Puncturing tool	Direction of puncture	Procedure related complications
Singer [1]	60	Rigid EsS	Transillumination & palpation	Custom troacar	Tr → Es	None
Brown [8]	> 160	Not required	Palpation	Scalpel	Tr → Es	None
Schipper [7]	12	Rigid EsS	No info	Dedicated troacar	Es → Tr	None
Lichtenberger [2]	3	Surgical LS	Inspection & palpation	Needle + metal cone	Es → Tr	None
Shaw [9]	4	Surgical LS	Transillumination	KTP laser	Es → Tr	None
LeBert [3]	39	Flexible EsS	Transillumination	Scalpel No. 11	Tr → Es	None
Pagedar [6]	6	Rigid EsS	Transillumination	Needle 15-gauge	Tr → Es	None
Damrose [5]	34	Rigid or flexible EsS	Transillumination & palpation	Needle 16-gauge	Tr → Es	1/34 retropharyngeal abscess, attributable to the rigid EsS

*n* number of patients, *EsS* esophagoscope, *LS* laryngoscope, *Es* esophagus, *Tr* trachea

## Methods

**Fig. 1:**
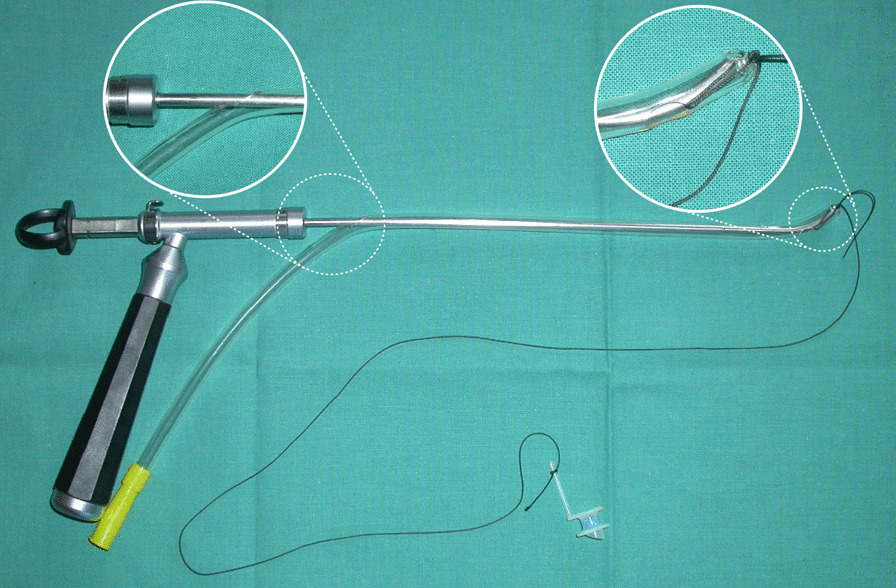
The Nelaton catheter securely loaded on the Lichtenberger endo-extralaryngeal needle carrier

The current study obtained the ethical approval from the Faculty of Medicine at Medical University "Prof. Dr. Paraskev Stoyanov"—Varna, Bulgaria (Protocol 026-14/23.05.2017). Written informed consent was not needed. All procedures contributing to this work comply with the ethical standards of the relevant national (Bulgaria) and institutional guidelines on human experimentation and with the Helsinki Declaration of 1975, as revised in 2008. Our modified technique for secondary TEP is based on EENC [2]. Preoperatively a thread (e.g. 1 UPS) is mounted on the tip of a 20 CH Nelaton catheter. An opening is cut into the catheter’s wall to insert the EENC, so the tip of the catheter can be stretched on the tip of the instrument. The thread should be placed on the back side of the instrument—protected by the catheter/carrier from the scalpel (Fig. 1).

Preoperatively all patients underwent lateral video fluoroscopic swallowing exam (VFSE) to check for stenosis of the neopharynx, evaluate the best potential TEP site and estimate the length of the prosthesis required.

The operation is performed under general anesthesia with intermittent apneic ventilation. Perioperatively patients were given 80 mg gentamycine i.v. as a prophylaxis of eventual infections.

**Fig. 2:**
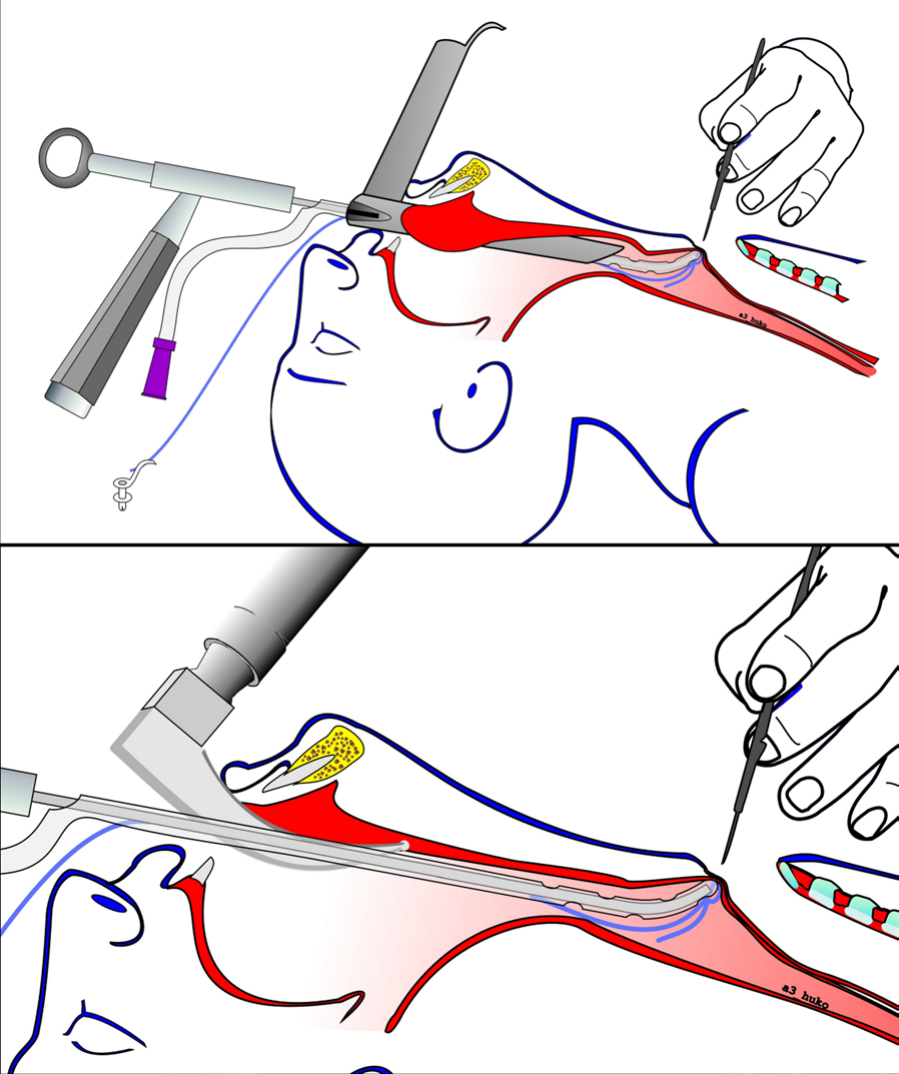
The intervention can be carried with both Kleinsasser type surgical laryngoscope (top) or intubational laryngoscope of the Macintosh type (bottom). The tip of the endo-extralaryngeal needle carrier is positioned by digital palpation through the stoma. The puncture is performed with scalpel blade 11

We used two different approaches for introducing the needle holder. The first one involves the use of a rigid standard surgical endoscope Kleinsasser type (Fig. 2 top). Later the technique was further simplified—only an intubational laryngoscope of the Macintosh type was used (Fig. 2 bottom). With both approaches no direct visualization of the puncturing site was required or aimed, but only of the entry of the esophagus.

**Fig. 3:**
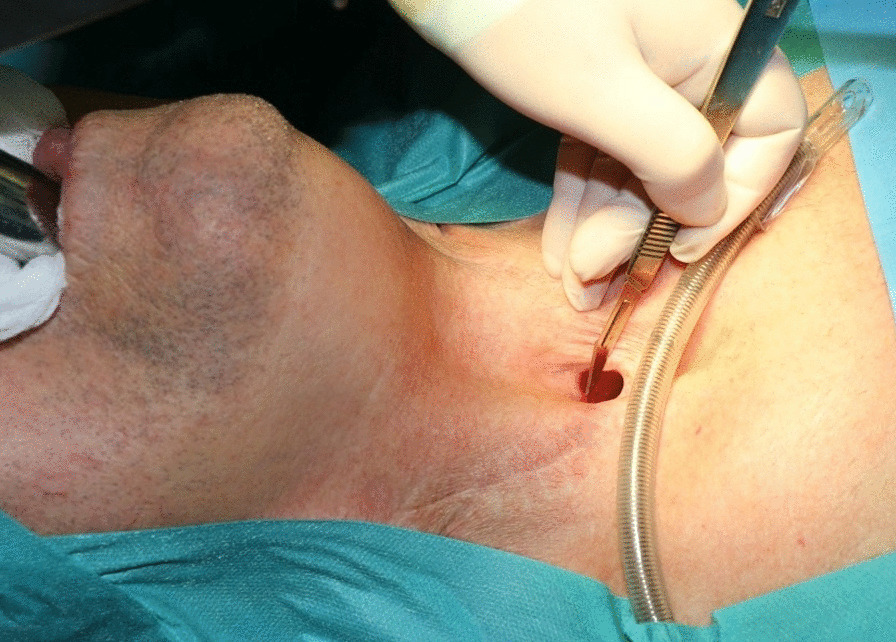
Intraoperative view of the bulging at the tip of the instrument. The EENC is introduced via Kleinsasser type surgical laryngoscope. The manipulations/incision are performed under intermittent apneic ventilation

As in any TEP the keypoint here is the selection of the puncturing site. Digital palpation against the catheter/carrier tip allows evaluation of the local tissue stiffness and thickness. The endo-extralaryngeal needle holder is further slightly pushed to clearly bulge the TEP site. Upon defining the position, a 3–4 mm stab incision with a scalpel blade 11 is made in the posterior tracheal wall to the catheter tip (Fig. 3).

Further minor dissection may be needed with a curved dissecting forceps (e.g. mosquito) till the tip of the catheter shows in the tracheal lumen. The thread is detached from the catheter and brought through the incision with gentle pull with the forceps. The EENC is removed together with the catheter. The tail of the voice prosthesis is fixed on the thread at the patient’s mouth. A gentle transstomal pull on the thread brings the prosthesis through the mouth and the neopharynx in a retrograde fashion and so it is inserted into the puncture site.

In the postoperative period patients are allowed to resume liquids and a soft diet, with advancement of diet over the next 1–3 days as tolerated by the patient. Voicing is permitted 2–3 days later.

## Results

This novel TEP technique was used in 14 male patients (mean age 60.6 ± 6.1 years, range 50–70 years). All patients were subjected to laryngectomy for primary laryngeal squamous cell carcinoma. Three of the patients had undergone unilateral and one—bilateral neck dissection associated with the laryngectomy. One of the patients had had pharyngocutaneous fistula with secondary healing. All patients had received postoperative radiation with mean total radiation doses 58 Gy (56–60 Gy). A total of 18 in-clinic secondary TEPs with immediate retrograde voice prosthesis placement were performed, all under general anesthesia. Mean time interval from total laryngectomy and first TEP ranged from 4 to 32 months. Three patients underwent the procedure after closure of a previous TEP (1 patient with three procedures, 2 patients with each two procedures). In four (first) interventions the neopharynx was exposed with a standard rigid scope Kleinsasser type. In the rest 14 interventions the EENC was introduced using intubational laryngoscope of the Macintosh type. In no case direct visualization of the puncture site was aimed or achieved. An InHealth Technologies indwelling voice prosthesis was placed in 15 interventions, and an ATOS Medical Provox 3 indwelling voice prosthesis in the remaining 3 interventions. There were few instances of the prosthesis being pulled through, requiring to start the procedure again. Prosthesis placement was successful in all but one case. This patient failed to pass air through the shunt on the first postoperative day. VFSE revealed the esophageal flange of the prosthesis was malpositioned within the channel of the tracheoesophageal fistula/the soft tissues at a distance from the contrast delineated lumen. The patient underwent repositioning of the prosthesis the same day again under general anesthesia. In this case only complementary flexible esophagoscopy was used to verify the optimal position of the esophageal flange. This early reintervention is not counted as another TEP.

In all cases there was only minor bleeding, none required use of cautery or other methods of bleeding control apart of suction and temporary compression with the cuff of the tracheal tubus. No other early or late complications were observed. The mean follow-up time was 14 months with no other early or late TEP or prosthesis related problems observed.

## Discussion

Since the last decades of the twentieth century the rehabilitation of voicing in laryngectomees relays on Blom-Singer puncture and implantation of voice prosthesis [1]. Some variations include the use of flexible transoral and transnasal esophagscopy for secondary TEP, or even retrograde passage of a gastroscope via mini laparotomy and gastrostomy [3, 4]. Generally two major types of TEP puncturing devices are used—needle-shaped troacars with conical dilators [2, 5–7] or scalpel blades [3, 4, 8]. Lasers have also been tried [9].

The majority of the TEP associated complications are related to the rigid endoscopy [10]. The TEP techniques, which avoid the use of a rigid scope seem advantageous in this particular aspect [2, 8–11]. Thus we deliberately adopted the Lichtenberger technique at our institution. The original endo-extra esophagotracheal technique described by Lichtenberger uses an EENC and a specially designed ETP-set, consisting of a metal cone with a puncturing tip and a counterfixing pierced ball [2]. In our hands the progressive dilating of the tissues with the cone appeared difficult, requiring important pull and thus quite traumatic for the surrounding tissues. Scared stiff tracheoesophageal junction behind the stoma is even more difficult to puncture, the needle tends to slide away from the predefined puncture site and the dilators are pulled through with inappropriate force. This is what made us abandon the original Lichtenberger technique and switch to the use of conventional scalpel and dissector technique similar to Evans [8].

The VFSE was used to check for stenosis or diverticula of the neopharynx, evaluate the best potential TEP site and estimate the length of the prosthesis. Though VFSE is often not standard of care for laryngectomees, it is incorporated in the routine work in our department and is found by us useful in the management of the early and late postoperative period in LE patients.

The major advantages of our approach for TEP include:

1. No rigid esophagoscopy, thus reducing the risk of trauma to the mucosa and related complications. No direct visualisation of the TEP site through the esophagus is needed neither during placement of the prosthesis, nor for verification of its internal flange. This may be especially helpful in cases with narrow esophagus, solid scar behind the stoma, tortuous passage and compromised mobility of the neck spine. Still in one case initial malposition of the prosthesis was suspected and verified by VFSE. During the repositioning complementary flexible esophagoscopy proved useful.
2. No dilating troacar for the tissues is used at the TEP site, compared with the original Lichtenberger technique. The systemic disadvantage of using a dilator or troacar as a main tool is causing excessive blunt trauma to the surrounding tissues that is hard to control and is disproportional to the positive effect of the puncturing itself. An incision with a sharp blade is much less traumatic and can be precisely tailored in small increments till the tip of the catheter shows. The belief, that the use of a scalpel to create the opening may be disadvantageous in terms of risk for leakage and the possibility of enlargement of the fistula has not been proven by any study/series. Additional use of curved dissecting forceps may be necessary only occasionally. Bleeding in our series was minor and did not necessitate cautery.
3. The original Lichtenberger procedure is simplified by the use of “routine” instruments and disposables (a blade, a dissector and a catheter), that are readily available in any operating room and do not require special skills or training form the surgeon. Still the Lichtenberger EENC is very handy allowing for easy introduction, firm hold and precise rotational and longitudinal orientation of the tip at the TEP site. The intubational laryngoscope of the Macintosh type seems to be easier to operate with and also less traumatic to the mouth and neopharynx, compared to the standard rigid surgical endoscope of Kleinsasser type. The curvatures of the Macintosh blade and the Lichtenberger EENC appear to be advantageous in patients who do not have enough head extension because of neck fibrosis, where a straight rigid scope is difficult to insert.
4. The Nelaton catheter provides a soft shield to the tip of the EENC. Thus the trauma of the metal instrument tip to the hypopharyngeal and esophageal mucosa is further minimized during insertion and positioning, which seems a common drawback of the Lichtenberg and Evans' forceps technique [8].

## Conclusions

We present a novel approach for the creation of a secondary TEP for voice acquisition based on the Lichtenberger EENC. It is precise, safe, and easier to perform than the original technique. The switch from dilating to cutting instruments is especially advantageous in thick scared tissues. Although our experience is limited, it is believed, that the soft blunt tip of the Nelaton catheter is superior to both bare tip Lichtenberger EENC [2] and Evans’ modified abdominal/gynecological forceps [8] in terms of ease of introduction through stenotic or tortuous neopharynx/esophagus, safety for the esophageal mucosa and as a semielastic mechanical opposition to the scalpel blade. Outcomes and complications have proven favorable.

## Data Availability

The datasets used and/or analyzed during the current study are available from the corresponding author on reasonable request.

## Abbreviations

EENC: Endo-extralaryngeal needle carrier
Es: Esophagus
EsS: Esophagoscope
LS: Laryngoscope
n: Number of patients
TEP: Tracheoesophageal puncture
Tr: Trachea
VFSE: Video fluoroscopic swallowing exam
